# Crizotinib and Surgery for Long-Term Disease Control in Children and Adolescents With ALK-Positive Inflammatory Myofibroblastic Tumors

**DOI:** 10.1200/PO.18.00297

**Published:** 2019-05-16

**Authors:** Toby Trahair, Andrew J. Gifford, Ashleigh Fordham, Chelsea Mayoh, Mitali Fadia, Robyn Lukeis, Andrew C. Wood, Santosh Valvi, Roderick D. Walker, James Blackburn, Erin E. Heyer, Tim R. Mercer, Draga Barbaric, Glenn M. Marshall, Karen L. MacKenzie

**Affiliations:** ^1^ Sydney Children’s Hospital, Randwick, New South Wales, Australia; ^2^ Children's Cancer Institute, Sydney, New South Wales, Australia; ^3^ University of New South Wales, Sydney, New South Wales, Australia; ^4^ Prince of Wales Hospital, Randwick, New South Wales, Australia; ^5^ Canberra Hospital, Garran, Australian Capital Territory, Australia; ^6^ Australian National University Medical School, Acton, Australian Capital Territory, Australia; ^7^ St Vincent’s Hospital, Darlinghurst, New South Wales, Australia; ^8^ University of Auckland, Auckland, New Zealand; ^9^ Perth Children’s Hospital, Perth, Western Australia, Australia; ^10^ Queensland Children’s Hospital, South Brisbane, Queensland, Australia; ^11^ Garvan Institute of Medical Research, Sydney, New South Wales, Australia; ^12^ Altius Institute for Biomedical Sciences, Seattle, WA; ^13^ Children’s Medical Research Institute, Westmead New South Wales, Australia

## Abstract

**PURPOSE:**

Before anaplastic lymphoma kinase (ALK) inhibitors, treatment options for *ALK-*positive inflammatory myofibroblastic tumors (AP-IMTs) were unsatisfactory. We retrospectively analyzed the outcome of patients with AP-IMT treated with crizotinib to document response, toxicity, survival, and features associated with relapse.

**METHODS:**

The cohort comprised eight patients with AP-IMT treated with crizotinib and surgery. Outcome measures were progression-free and overall survival after commencing crizotinib, treatment-related toxicities, features associated with relapse, outcome after relapse, and outcome after ceasing crizotinib.

**RESULTS:**

The median follow-up after commencing crizotinib was 3 years (range, 0.9 to 5.5 years). The major toxicity was neutropenia. All patients responded to crizotinib. Five were able to discontinue therapy without recurrence (median treatment duration, 1 year; range, 0.2 to 3.0 years); one continues on crizotinib. Two critically ill patients with initial complete response experienced relapse while on therapy. Both harbored *RANBP2-ALK* fusions and responded to alternative ALK inhibitors; one ultimately died as a result of progressive disease, whereas the other remains alive on treatment. Progression-free and overall survival since commencement of crizotinib is 0.75 ± 0.15% and 0.83 ± 0.15%, respectively.

**CONCLUSION:**

We confirm acceptable toxicity and excellent disease control in patients with AP-IMT treated with crizotinib, which may be ceased without recurrence in most. Relapses occurred in two of three patients with *RANBP2-ALK* translocated IMT, which suggests that such patients require additional therapy.

## INTRODUCTION

Inflammatory myofibroblastic tumors (IMTs) are rare and predominantly arise in the abdomen, pelvis, and chest. Approximately 40% to 60% of IMTs express anaplastic lymphoma kinase (ALK) and have associated *ALK* gene rearrangements.^1-4^ Multiple *ALK* fusion partners have been identified, including, but not limited to, *CARS*,^5^ *TPM3*, *TPM4*,^2,6^ *CLTC*,^7,8^ *RANBP2*,^9^ *SEC31L1*,^10^ *PPFIB1*,^11^ *FN1*,^12^ *IGFBP5*, *THBS1*,^13^ *DCTN1*, *RRBP1*, *TFG*,^6^ and *EML4*.^14^ Cryptic translocations that involve *ROS*, *ETV6*, and *NTRK* also have been identified in ALK-negative IMT.^15-18^ Currently, a poor understanding exists of how the *ALK* fusion partner influences the clinical behavior of IMT, except in cases of epithelioid inflammatory myofibroblastic sarcoma (eIMS), a clinically aggressive tumor associated with a high mortality before the introduction of ALK inhibitors (ALKis).^19,20^ eIMS is characterized by perinuclear or nuclear membrane ALK staining^6,19,21^; CD30 expression^19,20,22,23^; and characteristic ALK fusion partners, namely *RANBP2*,^19,20,23^ *RRBP1*,^6^ and *EML4*.^14^

**CONTEXT**

**Key Objective** To document the long-term outcome of a cohort of children and teenagers diagnosed with widespread, multifocal, ALK-positive inflammatory myofibroblastic tumors (AP-IMTs) treated in a crizotinib compassionate use access program.
**Knowledge Generated** Crizotinib and surgery are effective for widespread AP-IMTs in most patients who are able to stop treatment safely. Although well tolerated, recurrent crizotinib adverse effects, including skin infection and fractures, were observed and contributed to decisions to cease therapy. Disease control in the aggressive IMT variant *RANBP2-ALK*–rearranged epithelioid inflammatory myofibroblastic sarcoma was suboptimal, with early, on-treatment relapses.
**Relevance** Many patients with AP-IMT have excellent outcomes with crizotinib and surgery, which confirms that crizotinib should be considered as first-line therapy for patients with widespread or unresectable disease. The poorer outcomes in patients with *RANBP2-ALK*–rearranged epithelioid inflammatory myofibroblastic sarcoma suggests that specific patient subgroups, defined by pathology and *ALK* translocation partner, could benefit from intensified or combination therapy.

Surgery remains central to IMT therapy. Surgical resection with clear margins results in excellent outcomes.^24,25^ Although incomplete resection is associated with recurrence, favorable outcomes have been reported with adjuvant chemotherapy.^24-26^ ALKis have been shown to be safe and effective in *ALK-*rearranged tumors, including IMTs,^27-30^ but no randomized data have compared ALKi treatment and chemotherapy. Some authors continue to advocate surgery and adjuvant chemotherapy.^25^ To better define the utility of ALKi treatment in ALK-positive IMT (AP-IMT), we analyzed the outcome of a cohort of eight patients with AP*-*IMTs treated with crizotinib. We describe the treatment response, patient outcomes, and treatment-related toxicities associated with crizotinib treatment.

## METHODS

### Participants and Treatment

The cohort comprised eight patients diagnosed with AP-IMT from 2009 onward and who were treated with crizotinib. Patients were treated at four children’s cancer centers, including Sydney Children’s Hospital (n = 5), Queensland Children’s Hospital (n = 1), Princess Margaret Hospital (n = 1), and Starship Children’s Hospital (n = 1). The cohort includes all patients diagnosed with AP-IMT from 2009 onward. The diagnosis of AP-IMT was established locally with confirmation of ALK overexpression by immunohistochemistry (IHC). *ALK* locus rearrangement was detected using fluorescence in situ hybridization (FISH) with a dual-color break-apart probe (Vysis; Abbott Laboratories, Abbott Park, IL).

Crizotinib was provided by Pfizer (New York, NY) using a noncommercial supply management system for *ALK-*rearranged malignancies. Eligibility criteria for patients with AP-IMT were based on version 6.0 of the Pfizer Named-Patient Early-Access Guidance dated November 1, 2011, and included a diagnosis of an AP-IMT, adequate organ function, stable neurologic disease, and no clinical or laboratory evidence of significant cardiorespiratory disease.^31^ For AP-IMT, demonstration of ALK positivity by IHC alone was sufficient. Crizotinib was used at the pediatric maximum tolerated dose (280 mg/m^2^/dose twice a day orally).^28,29^ A treatment cycle was defined as 28 days, and crizotinib was continued at physician discretion while there was evidence of clinical benefit. The first patient commenced crizotinib in 2013, with the clinical outcomes reported to December 2017. Guidelines for safety monitoring; lifestyle recommendations, including contraception; management of adverse events (AEs); dose modification for toxicity and AE reporting; grading; and attribution were as recommended in the Pfizer Crizotinib Named-Patient Early-Access Reference Guide.^32^ Briefly, recommended clinical monitoring for safety included monthly blood tests, including a full blood count and liver function tests, for the first 3 months then repeated as clinically indicated. It was recommended that AEs be identified throughout the entire course of treatment with grading, causality, and reporting to the Pfizer drug safety unit within 24 hours of identifying each AE. Disease staging and treatment response were determined locally by clinical examination and laboratory assessment combined with anatomic (computed tomography [CT], magnetic resonance imaging, and/or ultrasound) and functional (positron emission tomography [PET]/CT scan) imaging. Imaging modality and the timing of disease response reassessments were at the discretion of individual clinicians. Response assessment was based on local report supported by imaging reports with tumor measurements. Imaging and response assessment were not centrally reviewed. Single diameter measurements were used to quantify treatment response on the basis of Response Evaluation Criteria in Solid Tumors (RECIST) version 1.1.^33^ A complete response (CR) was defined as disappearance of all lesions, partial response (PR) as a 30% or greater reduction in the diameters of all lesions with no new lesions, progressive disease (PD) as a 20% or greater increase in the diameters of all lesions or the appearance of new sites of disease, and stable disease as no evidence of significant change in all lesions to qualify as PD, PR, or CR.

Outcomes and toxicity were analyzed retrospectively. Toxicity guidelines data were graded according to the Common Terminology Criteria for Adverse Events (version 4). The primary outcomes of interest were progression-free survival (PFS) and overall survival (OS) since the commencement of crizotinib, the incidence of severe treatment-related toxicities (defined as grade ≥ 3), and features associated with treatment failure. PFS and OS were determined by Kaplan-Meier method using MedCalc version 18 (MedCalc, Ostend, Belgium) statistical software. The study was approved by the Sydney Children’s Hospital Network Human Research Ethics Committee (LNR/14/SCHN/90). The study was performed in accordance with the Declaration of Helsinki, and informed consent was obtained from the patients’ parents or legal guardians.

### Molecular Analyses

Tumor samples from the five patients treated at Sydney Children’s Hospital were subjected to RNA extraction for RNA capture sequencing to identify ALK fusion partners.^34^ Sequencing libraries were prepared from 1 μg of patient RNA using the KAPA Stranded RNA-Seq Library Preparation Kit (Roche, Basel, Switzerland). Capture was performed according to manufacturer’s instructions using custom biotinylated probes complementary to the coding regions of genes associated with chromosomal translocations in IMT. Captured libraries were sequenced to standard depth with 125-base pair paired-end sequencing using the HiSeq 2500 Kit v4 (Illumina, San Diego, CA). Sequencing reads were de-duplicated with Tally v15-065^35^ and adaptor sequences removed with Cutadapt v1.8.1.^36^ Postfiltering, reads were mapped to human reference genome hg38 with STAR v2.4.2a^37^ and fusions identified with STAR-Fusion and FusionCatcher v0.99.6 beta.^38^ Only the isoforms identified by both programs within each patient sample were used in additional analysis. Gene diagrams created to visualize the gene and fusion loci were based on GENCODE v.27 comprehensive exon annotation.

## RESULTS

### Clinical and Pathologic Features

**FIG 1.**
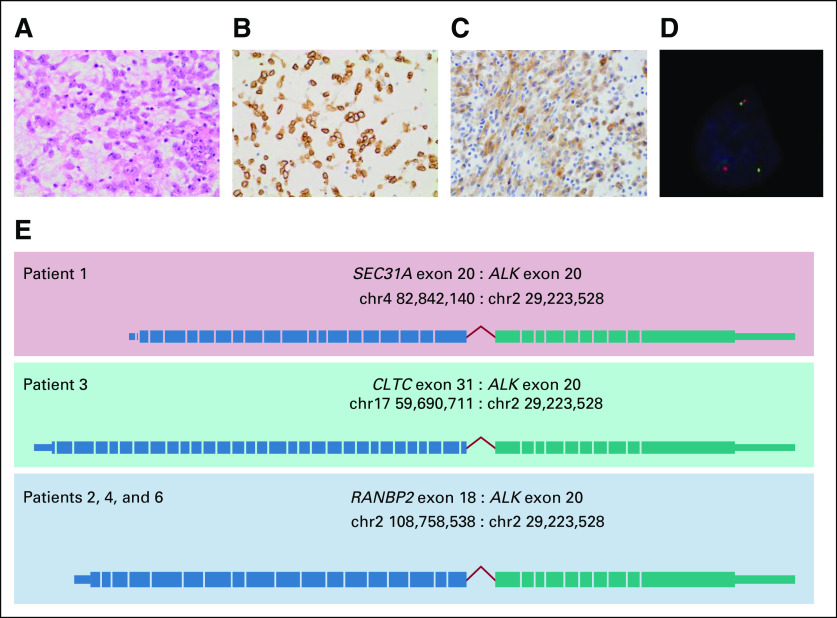
Histopathology of *RANBP2-ALK* epithelioid inflammatory myofibroblastic sarcoma and anaplastic lymphoma kinase (ALK) fusion partners identified in five patients with *ALK-*positive inflammatory myofibroblastic tumors. Histopathology of the resected ALK*-*positive epithelioid inflammatory myofibroblastic sarcoma tumor from patient 2 that demonstrates (A) epithelioid/rhabdoid tumor cells, (B) prominent perinuclear/nuclear membrane ALK staining on immunohistochemistry, (C) CD30^+^ tumor cells on immunohistochemistry, and (D) confirmation of an ALK translocation by fluorescence in situ hybridization using a dual-color break-apart probe (Vysis; Abbott Laboratories, Abbott Park, IL). The photograph demonstrates the ALK translocation (the split red and green signals in the lower portion of the image) and one normal ALK locus signal (red and green signals adjacent to each other in upper portion of the image) in a patient tumor cell. (E) Schematic representation of *SEC31A-*, *CLTC-*, and *RANBP2-ALK* fusions identified in five patients from Sydney Children’s Hospital by RNA capture sequencing.

The AP-IMT cohort comprised eight patients (four males, four females) diagnosed from 2009 to 2016 (Table 1). The median age at diagnosis was 7.1 years (range, 0.7 to 14.7 years). Two patients had significant prior medical histories, including a choledochal cyst and steroid-dependent nephrotic syndrome. The majority of AP-IMTs arose in the abdomen and pelvis (n = 7), with one thoracic tumor. Most patients (n = 5) had extensive disease with either multifocal or metastatic disease (Table 1). On the basis of the combination of perinuclear or nuclear membrane ALK IHC, CD30 positivity, and *RANBP2-ALK* translocation (identified in three patients), four patients were classified as having eIMS^19^ (Fig 1; Table 1). Tumor samples from five patients were subjected to RNA capture sequencing to identify *ALK* gene fusion partners. Three patients with ALK nuclear membrane staining were found to have *RANBP2-ALK* translocations, whereas two patients with diffuse cytoplasmic ALK staining were found to have *SEC31A-ALK* and *CLTC-ALK* fusions (Fig 1; Table 1).

**TABLE 1.**
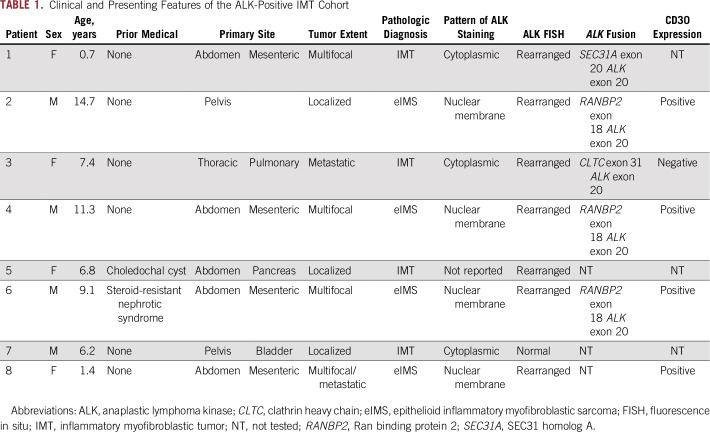
Clinical and Presenting Features of the ALK-Positive IMT Cohort

### Outcome After Treatment With Crizotinib and Surgery

The median time from diagnosis to commencing crizotinib was 19 days (range, 5 to 1,504 days; Table 2). Two patients were initially treated with conventional therapy. One patient (patient 1) with multifocal abdominal disease received long-term naproxen before developing progressive hepatic dysfunction. The other patient (patient 2) had a localized pelvic tumor that was completely resected. After tumor resection, the patient was treated with celecoxib but experienced a multifocal recurrence 2 months later. The remaining six patients were treated with crizotinib after confirmation of the diagnosis. One patient received prednisolone and a nonsteroidal anti-inflammatory drug for 5 days until crizotinib became available. Three patients were critically unwell and commenced crizotinib treatment in the intensive care unit (Table 2).

**TABLE 2.**
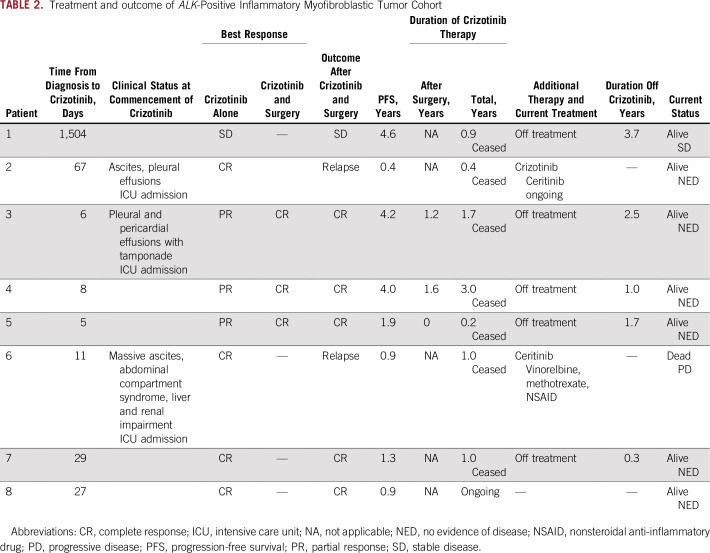
Treatment and outcome of *ALK*-Positive Inflammatory Myofibroblastic Tumor Cohort

**FIG 2.**
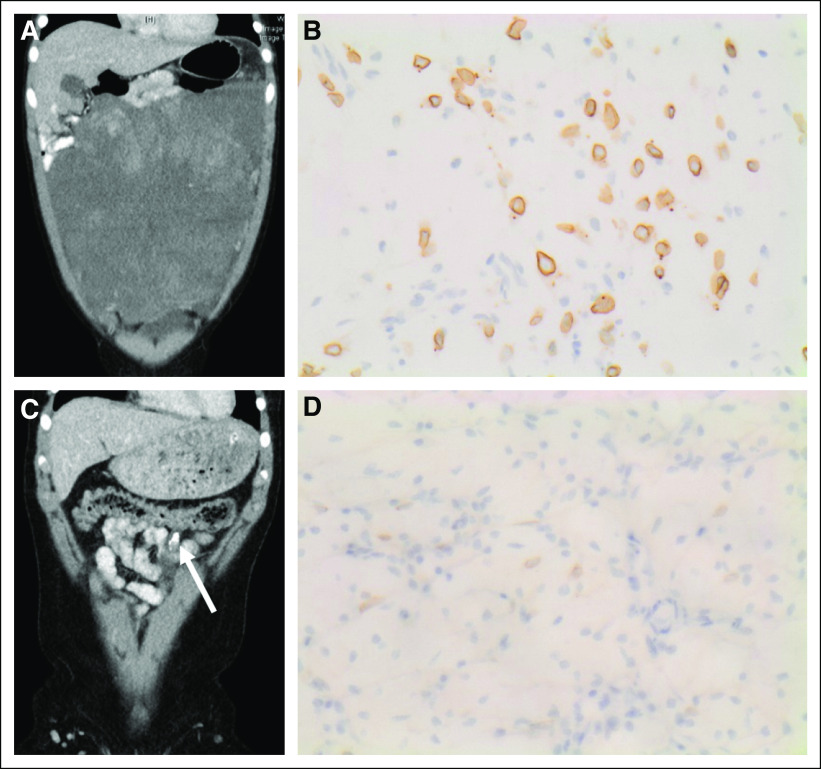
Treatment response and pathology in one patient with *RANBP2-ALK* rearranged epithelioid inflammatory myofibroblastic sarcoma. (A) Computed tomography scan at presentation demonstrates a large abdominal tumor in patient 4. (B) Anaplastic lymphoma kinase (ALK) immunohistochemistry on the tumor biopsy demonstrates prominent nuclear membrane/perinuclear ALK staining. (C) Computed tomography scan after crizotinib treatment shows almost complete shrinkage of the abdominal tumor with a small residual calcified mass (arrow). (D) ALK immunohistochemistry of resected residual tumor demonstrates frequent spindle-shaped cells with weak nuclear membrane/perinuclear ALK staining.

**FIG 3.**
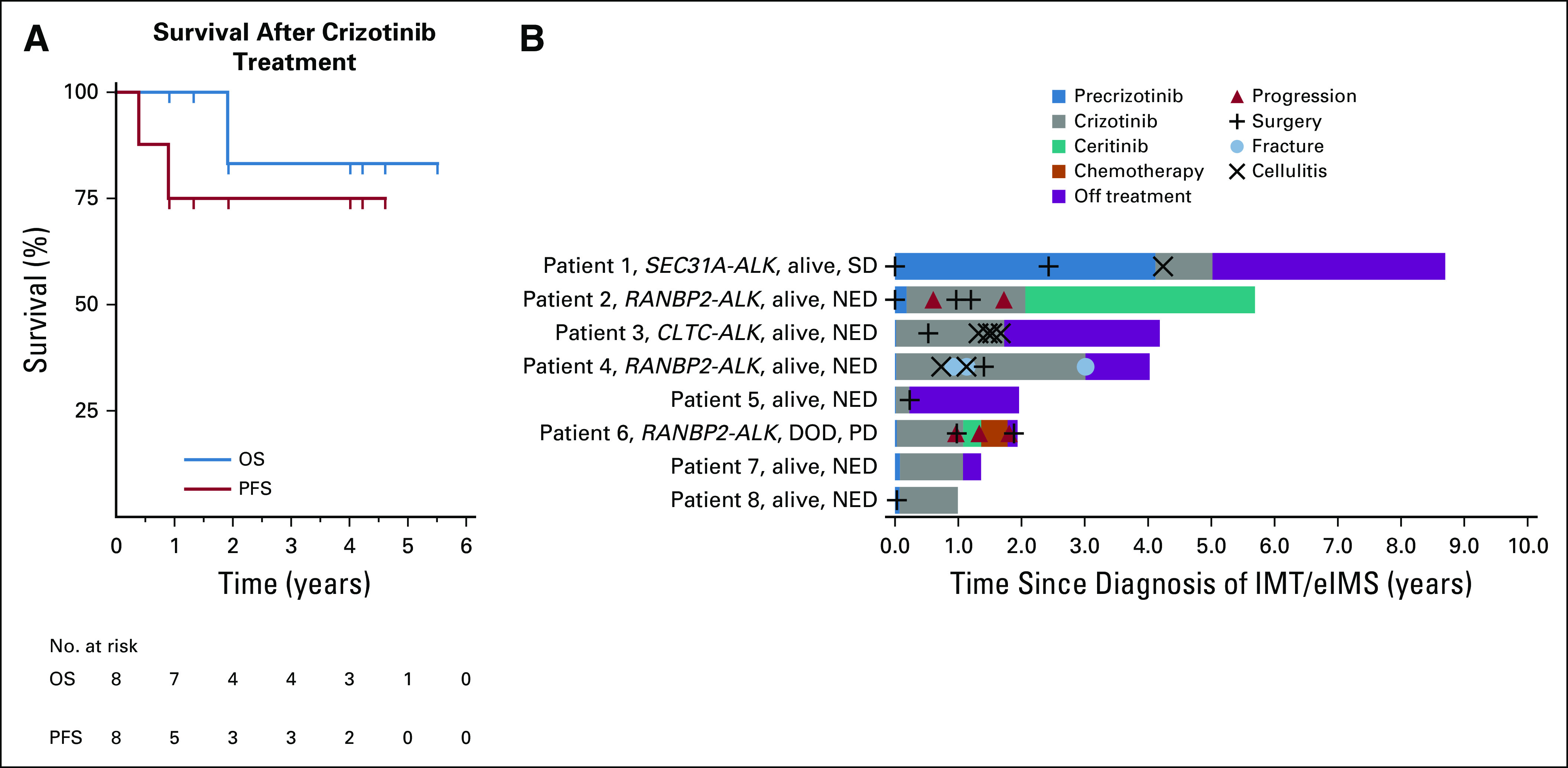
Survival analysis. (A) Kaplan-Meier analysis of overall survival (OS) and progression-free survival (PFS) since commencement of crizotinib treatment. (B) Swimmer plot analysis of individual patient outcomes since diagnosis, including treatment received and episodes of disease progression, surgery, cellulitis, and fractures. DOD, died as a result of the disease; eIMS, epithelioid inflammatory myofibroblastic sarcoma; IMT, inflammatory myofibroblastic tumor; NED, no evidence of disease; PD, progressive disease; SD, stable disease.

All patients had a clinical response to crizotinib (Table 2). In three patients, surgery was performed at the point of maximal tumor shrinkage to achieve a CR. Analysis of resected tumors showed extensive treatment-induced changes characterized by fibrous, hyalinized nodules, a patchy inflammatory infiltrate with foamy macrophages, and scattered foci of dystrophic calcification. In patient 4, plump spindle-shaped cells weakly staining for ALK-1 in a nuclear membrane/perinuclear pattern were identified in the resected residual tumor (Fig 2). Patient 1, who had multifocal abdominal and hepatic disease, had no evidence of tumor shrinkage on CT scan but had a complete metabolic response to crizotinib on fluorodeoxyglucose PET scan and normalization of abnormal liver function tests. Two of the eight patients, both with *RANBP2-ALK*–rearranged tumors, experienced disease relapse within the first year of crizotinib treatment despite initial CRs with crizotinib (Table 2; Fig 3). Five of the remaining six patients ceased crizotinib after a median treatment duration of 1 year (range, 0.2 to 3.0 years) and have been in stable PR or CR for a median of 1.7 years (range, 0.3 to 3.7 years). The 2-year Kaplan-Meier estimates of OS and PFS since commencement of crizotinib treatment are 0.83 ± 0.15% and 0.75 ± 0.15%, respectively (Fig 3).

### Toxicity of Crizotinib Therapy

Crizotinib was well tolerated (Table 3). The most common toxicity was asymptomatic neutropenia, which persisted until cessation of crizotinib (Table 3). In the first patient treated, the crizotinib dose was reduced from 280 to 200 mg/m^2^ as a result of asymptomatic grade 3 neutropenia. However, in subsequent patients, grade 3 neutropenia was managed with intermittent granulocyte colony-stimulating factor (5 μg/kg subcutaneous injection once or twice per week) without crizotinib dose reduction. There were seven episodes of cellulitis in three patients but no other documented bacterial or fungal infections. Crizotinib adverse effects contributed to decisions to cease treatment in two patients. Patient 3, who had preexisting mild, chronic dermatitis, experienced multiple episodes of cellulitis, and patient 4 experienced multiple fractures (Fig 3). Each patient had been in a prolonged remission, and the recurrent toxicity prompted the decision to cease crizotinib. In neither patient have there been additional fractures or episodes of cellulitis after ceasing crizotinib. Both patients remain well, off crizotinib, and in continuing CR.

**TABLE 3.**
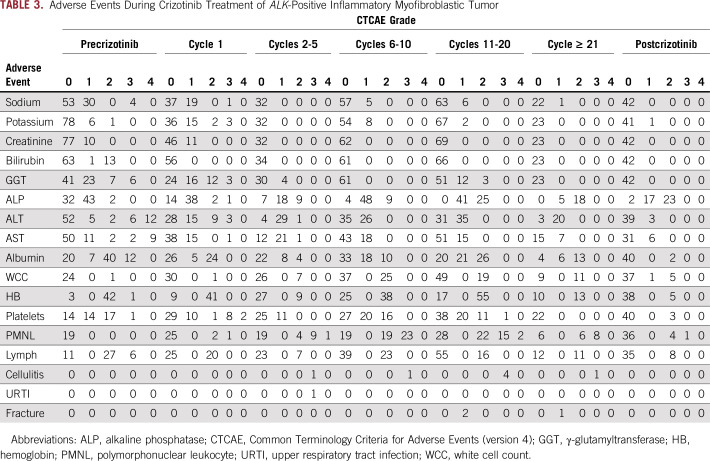
Adverse Events During Crizotinib Treatment of *ALK*-Positive Inflammatory Myofibroblastic Tumor

### Clinical Outcome After Recurrence on Crizotinib

Despite initial CRs, two patients with *RANBP2-ALK* eIMS experienced on-treatment disease recurrence after 4 and 10 months of crizotinib. The first patient (patient 2) experienced widespread abdominal and thoracic recurrence after crizotinib dose reduction for neutropenia. At recurrence, there were bilateral pleural effusions, large-volume ascites, multiple intra-abdominal peritoneal masses, and several lesions that indented the capsular surface of the liver. While undergoing disease re-evaluation, the crizotinib dose was increased to 280 mg/m^2^. There was a significant tumor response within 2 weeks of the dose increase, which was sustained at most disease sites and accompanied by resolution of pleural effusions, ascites, and peritoneal nodules. However, the hepatic capsular lesions did not resolve completely and were resected. The patient continued crizotinib at 280 mg/m^2^ for 1.4 years before experiencing additional local recurrences adjacent to the liver capsule, which were not amenable to resection. Crizotinib therapy was ceased, and the patient was changed to ceritinib.^39^ The patient went on to have a CR and is alive while continuing ceritinib without evidence of disease recurrence more than 3.5 years later. The second patient (patient 6), experienced a local recurrence adjacent to the liver 11 months after commencing crizotinib. An ALK p.I1171T mutation was identified after relapse on crizotinib.^40^ The patient was changed to ceritinib and achieved a partial tumor response of 2 months.^39,40^ After progression on ceritinib, the patient was commenced on low-dose chemotherapy (vinorelbine, methotrexate, and ketorolac),^41^ which resulted in a complete metabolic response on fluorodeoxyglucose PET scan, but with progressive calcification and no objective shrinkage on CT scan and magnetic resonance imaging. The tumor progressed after 6 months, and the patient died 23 months after the initial diagnosis.

## DISCUSSION

IMTs are rare tumors in children, teenagers, and adults commonly characterized by *ALK* gene rearrangement^15-18,42^ that are targetable with ALKis.^19,27-30^ Here, we present the clinical features, treatment, and outcome of a cohort of patients with AP-IMTs treated with crizotinib at the pediatric maximum tolerated dose.^28,29^ There are limitations to this study, being a retrospective analysis of outcome and toxicity in a compassionate access cohort. Eligibility was based on a standardized guideline, which included demonstration of ALK positivity on IHC, adequate organ function, and the absence of alternative treatment options as judged by the clinician. The cohort included all patients diagnosed with an AP-IMT at each of four participating centers. The access program provided guidance on safety monitoring and a requirement that clinicians identify, report, grade, and attribute causality for AEs within 24 hours of their identification. However, results were analyzed retrospectively. There was no central review of pathology or imaging results. The duration of crizotinib treatment was determined by individual clinician assessment of ongoing benefit for each patient. As a result, the data need to be interpreted with acknowledgment of these limitations.

The heterogeneous clinical features of AP-IMT are well represented and range from indolent disease to extensive tumors with life-threatening complications. Although *RANBP2-ALK*–rearranged eIMS has previously been identified as an aggressive AP-IMT subtype,^19,22^ we also observed life-threatening complications associated with a *CLTC-ALK*–rearranged IMT. Early consideration of an *ALK* rearrangement in the appropriate clinical context, particularly when patients present with extensive disease, is important for rapid diagnosis and timely initiation of ALKi treatment. This study demonstrates that crizotinib is tolerable and effective in patients with life-threatening complications, with three patients treated in the intensive care setting because of malignant effusions and one patient with associated hepatic impairment. Despite this, oral crizotinib was well tolerated and resulted in rapid clinical responses.

The phase I/II crizotinib trial conducted by the US Children’s Oncology Group (COG) demonstrated high response rates (CR, 36%; PR, 50%) with a favorable toxicity profile in children and teenagers with AP*-*IMTs.^28,29^ A lower objective response rate (50%) was observed in an adult crizotinib trial for AP-IMT.^30^ Our response data are concordant with the COG study, with all patients experiencing a clinical response. Factors that underlie differences in responses between pediatric and adult patients are not clear; however, the maximum tolerated dose in children (280 mg/m^2^)^28^ is higher than the adult dose of 250 mg (approximately equivalent to 140 mg/m^2^).^30^ At steady state, the pediatric dose (280 mg/m^2^) results in a 2.5-fold higher plasma area under the curve compared with the adult dose of 250 mg,^43^ which suggests that the response of AP-IMT to crizotinib may be partly due to plasma crizotinib levels. One patient with *RANBP2-ALK*–rearranged eIMS experienced disease recurrence after crizotinib dose reduction for asymptomatic neutropenia. A major, prolonged response occurred with an increased crizotinib dose, which suggests that achievement of adequate plasma crizotinib levels may be important to optimize response. We have no data, however, on plasma crizotinib levels. Analysis of crizotinib pharmacokinetics in the COG study suggested that at the pediatric maximum tolerated dose, free crizotinib plasma levels exceed those required for in vitro killing of *NPM-ALK*–rearranged anaplastic large-cell lymphoma (ALCL) cells.^43^

Tumor responses to crizotinib varied, with four patients (50%) achieving a CR, three (37.5%) a PR, and one (12.5%) stable disease. Surgical resection allowed two of the three patients with a PR to achieve a CR. One patient ceased crizotinib immediately after surgery, whereas the other two continued crizotinib for 1.2 and 1.6 years before cessation. One patient remains on crizotinib. The duration of crizotinib treatment was based on clinician preference and the circumstances of each individual patient. Of note, five (62.5%) of the eight patients ceased crizotinib without experiencing relapse or PD, which indicates that it is possible to cease crizotinib in AP-IMT without rapid disease recurrence as has been reported in ALCL.^44^ The toxicity profile observed was in line with the COG clinical trial.^28,29^ The most notable ongoing toxicity during crizotinib treatment was moderate to severe neutropenia, which was managed by intermittent granulocyte colony-stimulating factor. Skin infection has been reported in the COG study.^28^ Three patients experienced cellulitis, with two or more episodes occurring in two patients. Recurrent toxicity contributed to the decision to cease crizotinib in two patients, one because of multiple fractures (n = 3) and one because of multiple episodes of cellulitis (n = 4). Neither patient experienced additional fractures or cellulitis after ceasing crizotinib, which suggests that crizotinib likely contributed to these toxicities.

One patient had an AP-IMT by IHC, but the *ALK* FISH result was negative. Discordant ALK IHC and FISH results have been documented in non–small-cell lung cancer^45,46^ and recently in pulmonary AP-IMTs,^47^ where 19 of 24 AP-IMTs were FISH positive.^47^ In the five discordant cases, one had a *RET* rearrangement and the other four had either cryptic *ALK* fusions or expression of alternative *ALK* transcripts.^47^

The *ALK* fusion partners identified in this cohort (*RANBP2*, *CLTC*, and *SEC31A*) have been identified previously in IMT and other cancers, including acute myeloid leukemia,^48,49^ juvenile myelomonocytic leukemia,^50^ ALCL,^51^ congenital blastic plasmacytoid dendritic cell neoplasm,^52^ plasmacytoma,^53^ B-cell lymphoma,^54-60^ and lung adenocarcinoma.^61^ Recurrences occurred in two of three of the patients with *RANBP2-ALK* translocations, which suggests that this subgroup may be at higher risk of treatment failure despite initial CRs. Intensified treatment and/or combination therapy might be considered for patients with *RANBP2-ALK*–rearranged eIMS to reduce the risk of relapse or PD. In one patient with a *RANBP2-ALK* eIMS, ALK-positive cells were detectable in tumor resected after crizotinib treatment. Both patients who experienced relapse responded to ceritinib.^39^ One patient with small-volume disease at relapse continues to do well with ongoing ceritinib. The other patient who had bulky disease at relapse, experienced a PR with ceritinib before additional PD^39,40^ and death as a result of progressive cancer. An ALK p.I1171T mutation was identified from this patient’s tumor samples.^40^ The mechanism of treatment failure in the other patient who experienced recurrence has not been determined. Analysis of serial tumor samples may identify a resistance mutation in this patient as well. Mariño-Enríquez et al^19^ identified CD30 expression in *RANBP2-ALK* eIMS, a finding confirmed by others^20,22,23^ and in this cohort. CD30-directed therapy using brentuximab vedotin is effective in relapsed Hodgkin lymphoma and ALCL,^62,63^ a strategy that may be useful in patients with CD30^+^ *RANBP2-ALK* eIMS.

In summary, with acknowledgment of the limitations of our retrospective cohort study, the outcomes indicate that crizotinib and surgery are effective in long-term disease control for the majority of patients with AP-IMT. We suggest that most patients with unresectable AP-IMT be managed with crizotinib to the point of maximal tumor shrinkage followed by resection of residual tumor. Postoperative crizotinib should be considered in the context of incomplete resection, particularly for patients with eIMS, and can be safely stopped in many patients.
